# Research Progress and Prospects for Polymeric Nanovesicles in Anticancer Drug Delivery

**DOI:** 10.3389/fbioe.2022.850366

**Published:** 2022-02-11

**Authors:** Dan Li, Xi Zhang, Xiao Chen, Wei Li

**Affiliations:** ^1^ Cancer Center, The First Hospital of Jilin University, Changchun, China; ^2^ Department of Burn Surgery, The First Hospital of Jilin University, Changchun, China

**Keywords:** nanovesicle, anticancer drug, drug delivery, polymeric nanovesicles, cancer therapy

## Abstract

Polymeric vesicles served as the most promising candidates of drug delivery nanocarriers are attracting increasing attention in cancer therapy. Significant advantages have been reported, including hydrophilic molecules with high loading capacity, controllable drug release, rapid and smart responses to stimuli and versatile functionalities. In this study, we have made a systematic review of all aspects of nano-vesicles as drug delivery vectors for cancer treatment, mainly including the following aspect: characteristics of polymeric nanovesicles, polymeric nanovesicle synthesis, and recent progress in applying polymeric nanovesicles in antitumor drug delivery. Polymer nanovesicles have the advantages of synergistic photothermal and imaging in improving the anticancer effect. Therefore, we believe that drug carrier of polymer nanovesicles is a key direction for cancer treatment.

## 1 Introduction

Malignant tumors are a major problem threatening human health. Chemotherapy is one of the most important treatments for advanced malignant tumors (Bray et al., 2020). Chemotherapy refers to the use of one or more chemical drugs to inhibit tumor cell proliferation, infiltration and metastasis and ultimately kill cancer cells to cure the tumor. Many anticancer drugs (e.g., chemotherapy, biological therapies and hormones) have been developed to treat cancers; however, most chemotherapeutic drugs are not selectively distributed in tissues and have fast proliferation rates, thus affecting normal cells such as hair follicles, bone marrow and gastrointestinal tract cells. Hence, chemotherapy has serious toxic and adverse effects.

**FIGURE 1:**
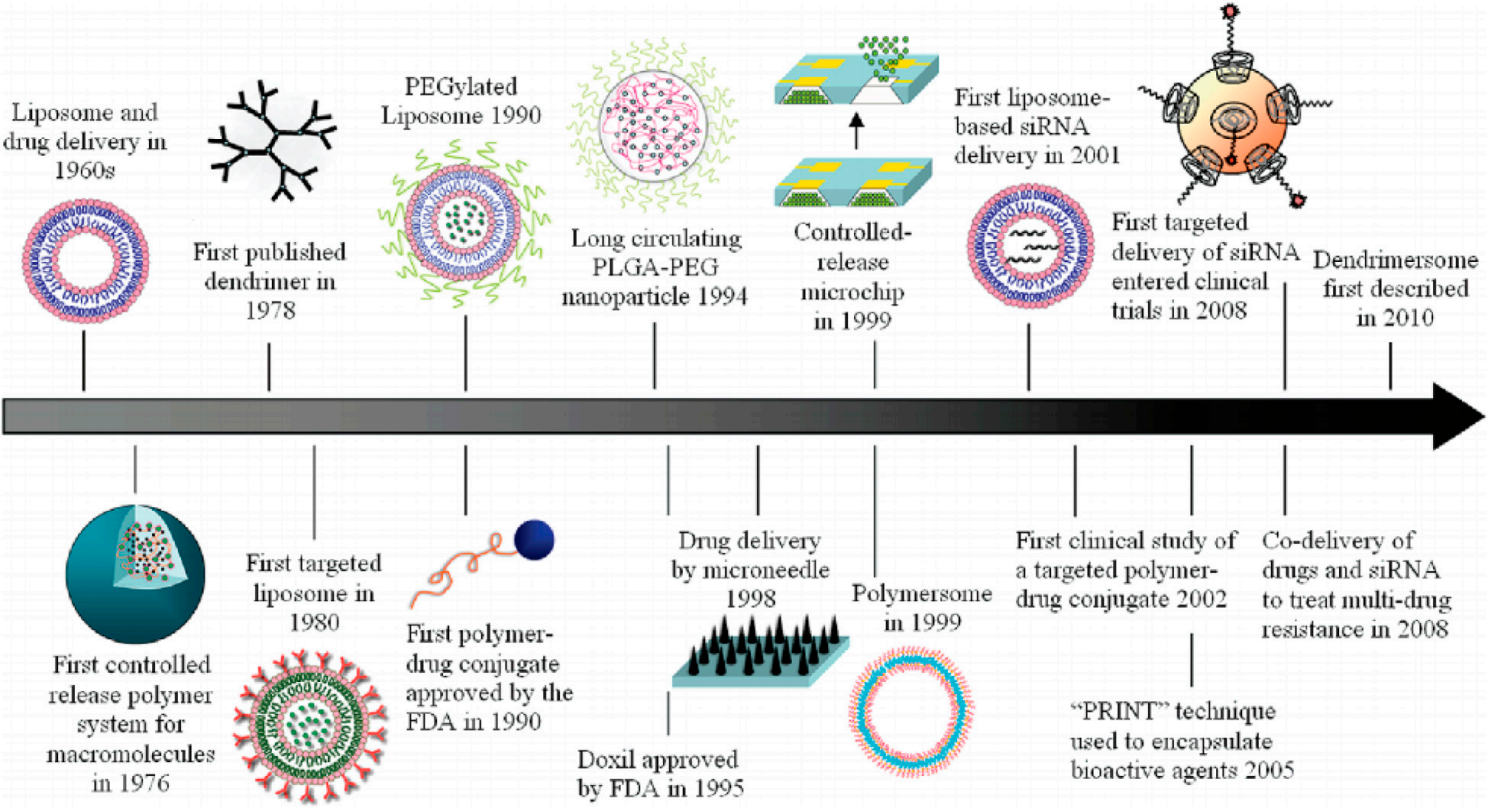
Historical timeline of major developments in the field of cancer nanomedicine. Reproduced with permission from Shi et al., 2010.

**FIGURE 2:**
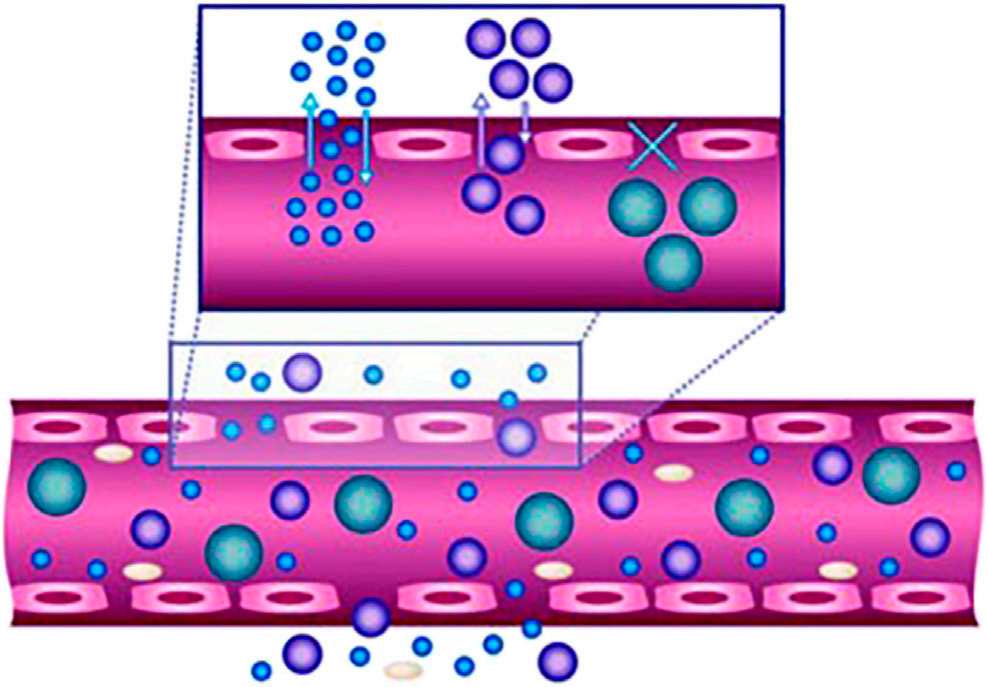
Efficacy of nanoparticles as delivery vehicles is highly size- and shape-dependent. The size of the nanoparticles affects their movement in and out of the vasculature, whereas the margination of particles to vessel wall is impacted by their shape. Reproduced with permission from Farokhzad and Langer, 2009.

Nanomedicines were first developed in the 1960s (Wang et al., 2008; Zhao et al., 2014; Bray et al., 2020) when scientists proposed the application of nanolipid vesicles (i.e., liposomes) for drug delivery. Since then, many nanodrug delivery systems have been developed. The main developments of nanomedicines can be summarized as follows (Wang et al., 2018) (Figure 1). In 1976, Langer et al. first proposed a sustained-release drug delivery system (Langer and Folkman, 1976), and in 1980, Yatvin et al. designed liposomes with pH-responsive drug release and active targeting functions for drug delivery (Yatvin et al., 1980). In 1986, Matsumura and Maeda proposed the enhanced permeability and retention effect (EPR effect) (Figure 2), stating that nanomedicines can gradually accumulate at tumor sites and remain in the tumor tissue based on the pathological characteristics of tumor vascular discontinuity and an incompetent tumor lymphatic system (Matsumura and Maeda, 1986). Langer and Folkman prepared the first long-circulating poly (ethylene glycol)-poly (lactic acid-ethanolic acid) nanoparticles, which were approved by the U.S. Food and Drug Administration (FDA) as the first nanomedicine for clinical use in treating ovarian cancer, metastatic breast cancer, and acquired immunodeficiency syndrome-related Kaposi’s sarcoma (Langer and Folkman, 1976). Since then, many nanomedicines have entered clinical trials, and some have been approved to clinically treat tumors. Currently, 15 nanomedicines have been approved by regulatory agencies such as the FDA and the European Medicines Agency for treating cancer, and over 50 nanomedicines are in clinical trials. The clinically approved nanomedicines include ten liposomes, two polymeric micelles, two nanoparticles, and one inorganic nanoparticle, but no polymeric nanovesicles have yet received clinical approval (Table 1).

**TABLE 1:** Approved cancer nanomedicines.

Nanomedicines	Approved Year	Formulation	Clinical application
Neocarzinostatin	1993	Polymer conjugates	Polymer conjugates
Doxorubicin	1996	Liposomes	Metastatic breast cancer
aclitaxel	2005	Albumin-bound paclitaxel nanoparticles	Advanced non-small-cell lung cancer
Vincristine	2012	Liposomes	Non-small-cell lung cancer
Cytarabine/daunorubicin	2017	Liposomes	Acute myeloid leukaemia
Paclitaxel	2017	Lipid nanoparticles	Advanced gastric cancer
None	2019	Hafnium oxide nanoparticles	Soft tissue sarcoma

**FIGURE 3:**
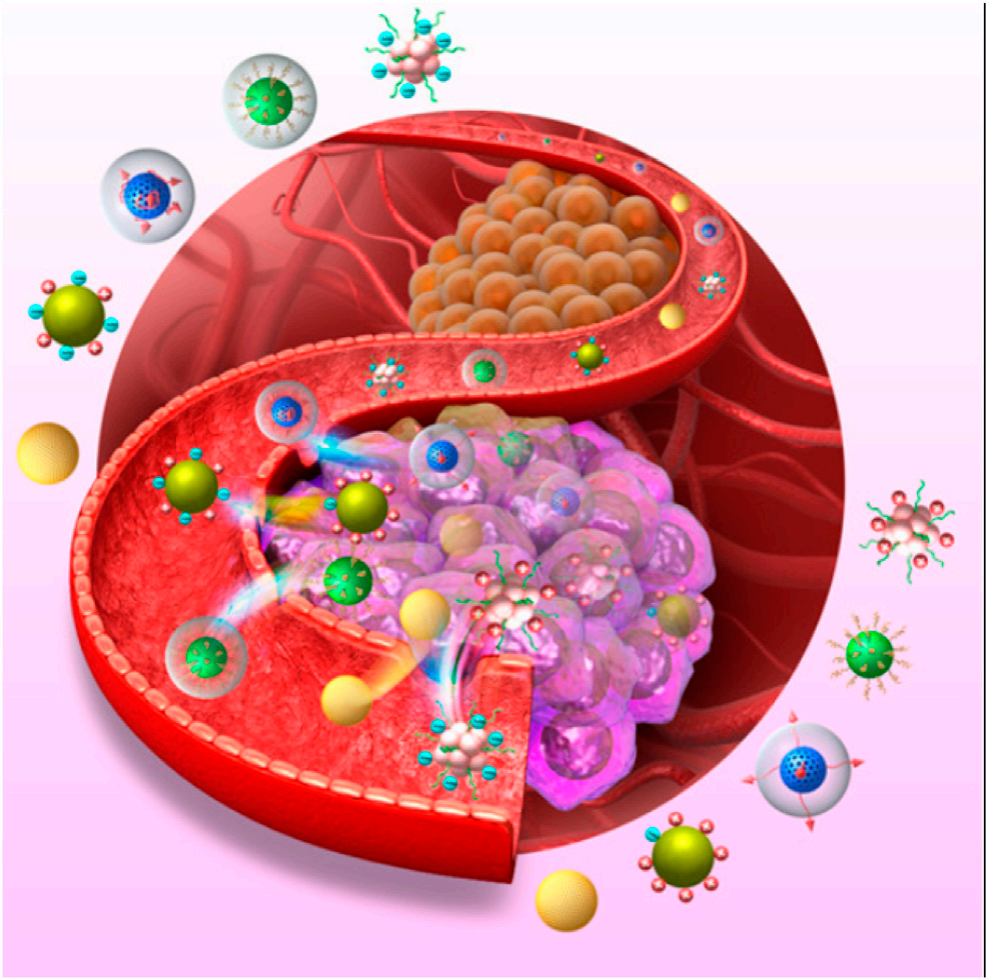
Various Nanocarries Systems Targeting Cancer Cells. Reproduced with permission from Jin et al., 2019.

Lipids, inorganic nanomaterials, and polymers are often used in nanocarrier systems (Figure 3). Lipids are used as raw materials to prepare lipid-based nanocarriers because they have excellent biosolubility and can wrap both hydrophilic and hydrophobic drug molecules. Some lipids can also attach drug molecules to the nanoparticle surface via adsorption to improve the drug’s ability to enter cancer cells by adsorption or cell membrane fusion, which significantly improves the drug’s bioavailability. Inorganic nanocarriers are easy to prepare and easy to control in size and shape. The special properties of inorganic materials, such as their optical, electrical and magnetic properties, provide a favorable basis for their use in targeted tumor therapy, diagnostic imaging and targeted drug delivery. Gold, mesoporous silica, and magnetic nanoparticles are used in inorganic nanodrug delivery systems. Polymer-based nanocarriers are the most widely studied and valuable system in clinical practice. The material composition and structure of polymeric carriers include linear polymeric carriers, polymeric micelles and self-assembled nanovesicles.

Polymer nanovesicles are self-assembled and formed by amphiphilic polymers in aqueous solution; they are similar to liposomes, with a closed membrane and ordered structure (Mikos et al., 1994; Jiang et al., 2015; Wang et al., 2015). The self-assembly morphologies of polymeric nanovesicles and micelles differ primarily in the hydrophilic and hydrophobic ratios of the polymers that make up the micelles or nanovesicles. Polymeric nanovesicles, such as liposomes, can be actively loaded with weakly alkaline or acidic hydrophilic drugs (e.g., doxorubicin hydrochloride) via ionic gradients (e.g., pH, ammonium sulfate, and calcium acetate gradient) and can be loaded with biomolecules via electrostatic or hydrogen bonding interactions. The thicker and tunable hydrophobic membranes of polymeric nanovesicles can be used to treat and diagnose cancer by loading hydrophobic drugs or fluorescent probes through hydrophobic interactions (Kim et al., 2009; Wang et al., 2015; Phan et al., 2021; Shreyash et al., 2021).

Despite the many advantages of polymeric nanovesicle carriers, no nanovesicular drugs have received FDA approval or are in clinical trials. Here, we review the progress in this emerging new drug system by first summarizing the properties of polymeric nanovesicles, then discussing their preparation methods. Next, we present the applications and recent research progress regarding polymeric nanovesicles as nanodrug carriers in therapeutics, including prolonged tumor retention, reversal of multidrug resistance, inhibition of tumor metastasis, prevention of tumor recurrence, and novel smart polymeric nanovesicle carrier antitumor drugs. Finally, we review the prospects and challenges of polymeric nanovesicles as nanocrushed carriers in cancer clinical therapy (He et al., 2019; Pearce and O’Reilly, 2019; Saha et al., 2019)

## 2 Characteristics of Polymeric Nanovesicles

Polymeric nanovesicles are vesicular cavity spheres or spheres with a liposomal bilayer structure formed via self-assembly of amphiphilic block copolymer molecules in water (Discher et al., 1999). The structure is specialized, with a hydrophilic inner cavity and hydrophobic membrane layer, while the outer surface of the membrane layer also has a hydrophilic shell, thus constituting a hydrophilic-hydrophobic-hydrophilic cavity-membrane-shell structure from the inside to the outside of the formation. Because the particle size can be controlled, polymeric nanovesicles offer both the advantages of nanoparticles and the unique advantages created by their specialized structure. Their characteristics are summarized below.

Polymeric nanovesicles are unique drug carriers with a hydrophilic lumen that can solubilize water-soluble active ingredients and a hydrophobic membrane layer that can solubilize lipid-soluble drugs. Polymeric nanovesicles can carry both these active ingredients simultaneously. The larger hydrophilic lumen facilitates encapsulation and protection of water-soluble biomolecules (e.g., drugs, peptides, proteins, enzymes, and RNA and DNA fragments) (Christian et al., 2009). The thicker hydrophobic membrane layer facilitates loading and solubilization of lipid-soluble drugs (e.g., paclitaxel) (Li et al., 2007). Polymeric nanovesicles do not need to change the structures of the drug molecules or biomolecules, such as proteins, to protect them from degradation and inactivation of their active ingredients, improve the drug stability, and enhance their efficacy and pharmacological properties.

Polymeric nanovesicles have a double membrane-layer structure that is similar to biological membranes and is more compatible with the drugs being delivered, thus making them good drug-delivery carriers *in vivo*. This double membrane layer can help the drug cross the biological barrier, change the drug’s distribution in the organism and reduce systemic toxic adverse effects (Santos-Magalhães and Mosqueira, 2010; (Xiong et al., 2011). The thicker double-layer structure of the vesicles increases the time needed for active molecules to enter and leave the vesicles, which can prolong the drug-release time, stabilize the blood concentration and improve the drug’s bioavailability. The hydrophilic shell structure of the outer layer enhances the spatial stability of the vesicles, prolongs the circulation time of the vesicles in the body, and further improves the drug’s bioavailability (Singh and Lillard, 2009; Alibolandi et al., 2016).

Because the particle size of polymeric nanovesicles is only a few hundred nanometers and has long circulation characteristics *in vivo*, drug-carrying vesicles can use the EPR effect of tumor cells to accumulate spontaneously through the permeable vascular tissue at the lesion site and obtain passive targeting ability (Photos et al., 2003). The EPR effect promotes selective enrichment of the nanovesicles and other macromolecular particles in cancerous tissues, which increases the blood concentration and improves the therapeutic efficacy. This increases the blood concentration of the drug at the site, improves the drug’s efficacy and reduces the systemic toxic adverse effects.

Specific ligands, such as antibodies, peptides, galactose, mannose and folic acid, can be stably attached to the surface of polymeric nanovesicles to confer active targeting functions and other biological effects on the vesicles. When receptors on the surfaces of target cells specifically recognize and bind ligands, the ligands can effectively mediate endocytosis vesicle into target cells and deliver more drug to the target cells while overcoming the nonspecific clearance of the reticuloendothelial system, improving the killing of target tissue cells, and reducing the toxic adverse effects to other cells.

Polymeric nanovesicles have good molecular designability. Selecting different amphiphilic polymers enables subjectively controlling the physicochemical properties (e.g., particle size, zeta potential, membrane thickness, elasticity, degradability, stimulus responsiveness, permeability, and drug-loading capacity) (Torchilin et al., 2003; Lin et al., 2006; Sutton et al., 2007; Danhier et al., 2010; Pawar et al., 2013) and the *in vivo* mode of action of the constructed vesicles. Polymeric materials with good biodegradability, whose degraded products are nontoxic or less toxic and can be excreted through normal physiological metabolism, are typically used to prepare vesicles to avoid long-term accumulation of toxic adverse effects. Differences in the tumor tissue and normal tissue microenvironments enable constructing vesicles using polymeric materials that respond to stimuli, which in turn makes the vesicles more responsive to stimuli (Zhang et al., 2012). These polymeric nanovesicles are stable when circulating *in vivo*, and when they reach the targeted lesion, they respond to a specific stimulus at that site by changing the properties of the polymeric chain segments and rapidly releasing their encapsulated drug or bioactive molecule at that specific site. Polymeric nanovesicles can respond to stimuli such as acidity, temperature, redox, enzymes, light, and magnetic fields. This enhances the targeting effect of the nanovesicles, enriches the vesicles at the target site, promotes cellular uptake of the vesicles and drug, improves the drug’s therapeutic effect, prevents toxic adverse effects to normal tissues or organs due to premature drug release, and prevents development of drug resistance due to slow release of the drug and nanovesicles at the target site.

## 3 Polymeric Nanovesicle Synthesis

Polymeric nanovesicles are commonly synthesized via film hydration, solvent volatilization, phase transfer, direct dissolution, electrical formation and microfluidic preparation (Liao et al., 2012; Shen et al., 2017).

In film rehydration, the amphiphilic polymer is completely dissolved in a volatile solvent, then the solvent is fully removed using a rotary evaporator to form a polymer film and vacuumed until completely dry. Water is then added to hydrate it. During hydration, water molecules penetrate the film, causing the hydrophilic chain segments to extend toward the aqueous phase, while the hydrophobic chain segments aggregate into a film layer and self-assemble into larger polymer nanovesicles.

In the solvent volatilization method (solvent evaporation), the amphiphilic polymer is fully dissolved in a volatile organic solvent, added dropwise to the aqueous phase while stirring, then continuously stirred to remove the organic phase, i.e., the polymer nanovesicles with a more uniform particle size distribution.

In the phase-transfer method (phase transfer), the amphiphilic polymer is fully dissolved in the organic solvent, which is miscible with water, then added dropwise to the aqueous phase while stirring to produce the polymer nanovesicles. This method allows subjectively regulating the size and distribution of the nanovesicles via the different solvents used.

In the dialysis method (dialysis), the amphiphilic polymer is fully dissolved in an organic solvent that is miscible with water, and the organic phase is purified by dialysis to obtain polymer nanovesicles.

In direct formation, amphiphilic polymers with a low glass-transition temperature are added to the aqueous phase and thoroughly mixed to produce the polymer nanovesicles. Water-soluble amphiphilic polymer materials that respond to external stimuli can be directly dissolved in the aqueous phase allowing the polymeric nanovesicles to self-assemble by controlling the pH or temperature of the aqueous phase.

Using electroformation, the polymer film is first formed on a cobalt wire electrode, then the electrode is combined with a Teflon shelf in a sealed chamber injected with sugar water. A suitable electric field is applied to the electrode, and the vesicles gradually form on the film surface after 15–60 min. After reducing the electric field intensity, the vesicles can be removed from the electrode.

Microfluidic fabrication is a newly emerging method for preparing polymeric nanovesicles. Compared with other methods, substances of large molecular weight can be effectively loaded to form large, uniformly distributed polymeric nanovesicles. This method uses the principle of W/O/W double emulsification to form a monodisperse W/O/W double-emulsified structure in a glass capillary, which is then placed under ventilation to allow the organic solvent to evaporate to obtain the polymeric vesicles (Lopresti et al., 2009).

## 4 Recent Progress in Applying Polymeric Nanovesicles in Antitumor Drug Delivery

**FIGURE 4:**
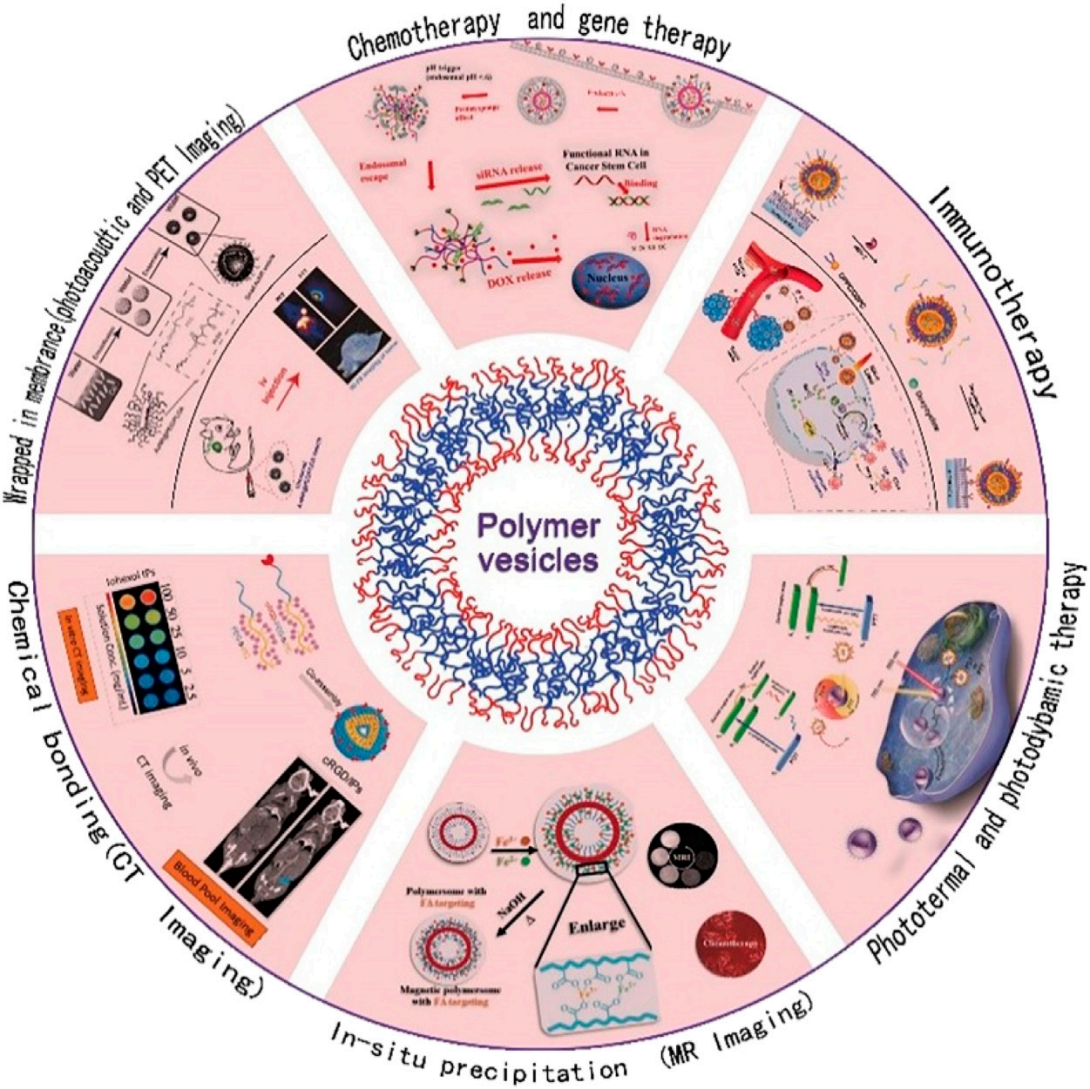
Recent progress in nanovesicle applications.

Polymeric nanovesicles are typically used as submicron hollow spherical structures, with the main structure composed of a polymer shell and hollow inner space (Tang et al., 2016). The nanovesicle cancer drug delivery system reduces adverse effects, improves treatment efficiency, and reduces the administration frequency (Figure 4). In this section, we introduce the latest progress in preparing nanovesicles and their applications as antitumor drug delivery carriers. We also introduce some newly emerging nanovesicles with potential for cancer drug delivery.

### 4.1 Research Progress on Polymeric Nanovesicle Preparation Methods

Using molecular design, Chen et al. synthesized the new amphiphilic trans- and *cis*-isomers, trans-PEG550-TPE-cholesterol (Chol) and *cis*-PEG550-TPE-Chol, and studied their self-assembly behavior. In these isomers, tetraphenylethylene (TPE) and Chol formed the hydrophobic portion, and ethylene glycol oligomer (PEG) formed the hydrophilic portion. *Trans*-PEG550-TPE-Chol self-assembled to form vesicles, while *cis*-PEG550-TPE-Chol tended to form cylindrical micelles. Under the same assembly conditions, both natural and artificial *cis*-trans mixtures (trans/cis)-PEG550-TPE-Chol self-assembled to form vesicles with nanopores. The trans-PEG550-TPE-Chol vesicles transformed into well-perforated vesicles and membrane structures after illumination. NMR results showed that part of the assembly of trans-PEG550-TPE-Chol was photoisomerized into its cis structure. Using the vesicles from the closed to the open state, these authors achieved controlled release of the encapsulated macromolecules (Chen et al., 2021)

Compared with conventional liposomes, polymer vesicles have strong stability and high chemical adjustability, but low membrane permeability; this significantly hinders the transport and exchange of substances through the vesicle membrane. To solve this problem, Hu et al. regulated the membrane permeability by embedding biological macromolecules and introducing stimulus-responsive components. However, most of these methods can cause vesicle disintegration or require complex chemical reactions to maintain the vesicle structure. In nature, orderly changes in the protein conformation of cells or organelle membranes can cause changes in membrane permeability, which in turn affects life activities such as molecular transport and apoptosis. However, regulating vesicular membrane permeability such as in biological systems is challenging for synthetic polymers. A new method for regulating the orderly transformation of polyamino acid conformation by using hydrophobic locking and oxidation gating has been proposed, in which the polymeric micellar-vesicle transformation is driven by conformation. Researchers obtained polyamino acid vesicles through rational design, and the polymer vesicle composition under the action of reactive oxygen species changed from *ß*-folding to *a*-helices, thereby reducing the membrane thickness and reconstructing the hydrogen bond and phase behavior. This enhanced the membrane permeability while retaining the vesicle’s integrity and enabled specific transmembrane transport of small molecules and macromolecular substances (Chenhao Hu et al., 2021).

Efficiently delivering genes or proteins and other biological macromolecules with therapeutic functions to cells is important in the field of biomedicine, and determining how to do this safely and efficiently poses a challenge. Cationic polymers are widely studied non-viral carriers, but forming stable and efficient nanoparticles with short chains, rigid small interfering RNA (siRNA) and proteins with low charge density and uneven distribution using common cationic polymers such as PEG-PLL is difficult. Although increasing the cationic charge density can solve this problem, a high charge density can significantly increase the cytotoxicity of the carrier. Zhou et al. found that single-stranded oligonucleotides with flexible chain structures and relatively small molecular weights could form complex nanovesicles with PEG-PLL to efficiently load various proteins and siRNA and transport them into cells. Vesicular F20some was constructed using functional oligonucleotide single-stranded F20 containing 20 fluorouracil units and PEG-PLL. The transport characteristics of the protein and siRNA *in vivo* and *in vitro* were systematically studied. F20 and synergistic siRNA or functional proteins were delivered simultaneously, yielding a nanopreparation with high antitumor activity. Using this method, single-stranded nucleotides can be used for various nucleotide sequences such as aptamers, CpG and microRNA; thus, it is a universal, efficient and minimally toxic delivery platform for macromolecules such as genes and proteins (Quan Zhou et al., 2021). However, improving the stability of the vesicles in a physiological environment is a focus of future research.

### 4.2 Recent Progress of Nanovesicles Cancer Drug Delivery Systems

#### 4.2.1 Real-Time Monitoring of Drugs can Be Achieved Through Synergistic Biological Imaging

Nanovesicles can achieve sustained drug release that can be monitored. Researchers have explored using porphyrin as a photosensitizer of nanovesicles for sustained drug release. Self-assembly of porphysome vesicles allows excellent drug-loading capacity and infrared absorption characteristics (Wang et al., 2008; (Lovell et al., 2011), and fabricated doxorubicin (DOX)-loaded supramolecular porphysome nanovesicles can be self-assembled using amphiphilic porphyrin derivatives. High concentrations of glutathione stimulate the release mechanism of DOX drugs, which is monitored through the fluorescence recovery of porphyrin derivatives (Xu et al., 2015) (Figure 1). Wu et al. prepared biomimetic nanovesicles equipped with DOX, which can be combined with photodynamics and chemotherapy, and achieved a tumor suppression rate of 91.6% (Tingting Wu et al., 2018). PEG-boron-dipyrromethene-based nanovesicles with both near-infrared imaging and drug delivery functions have been prepared and can be used for the dual purpose of imaging and treatment. These nanovesicles allow monitoring the drug-release law in real time (Tingting Wu et al., 2018).

Polypyrrole/UiO-66 metalloorganic framework nanoparticle-loaded arene-based pseudorotaxane nanovesicles were fabricated based on the above studies. After modifying polyethylenimine with folic acid-polyethylenimine, the nanocapsules are coated with 5-fluorouracil for near-infrared imaging and drug release. The effect was verified via *in vitro* experiments (Ming-Xue Wu et al., 2018).

### 4.2.2 Polymer-Based Hybrid Vesicle Delivery System

To solve the problem of multidrug resistance in metastatic tumors, various inorganic substances have been introduced into polymer nanovesicles. Song et al. developed heparin/protamine/calcium carbonate (HP/PS/CaCO_3_)-hybrid nanovesicles. In this treatment system, tariquidar, a drug-resistant inhibitor, is loaded into modified nanovesicles when assembled together with DOX in an aqueous medium. Calcium carbonate imparts pH sensitivity to HeLa and MCF-7 cells, and *in vitro* experiments on drug-resistant cancer cells (MCF-7/ADR) confirmed that this drug-carrying system improved the efficiency of killing drug-resistant tumor cells; thus, this may be a new treatment strategy for drug-resistant tumor cells (Gong et al., 2015) (Figure 2). Natural polymers have good biocompatibility (Park et al., 2003; Sarmento et al., 2006; Wang et al., 2014), but their hydrophilicity makes it difficult to encapsulate anticancer drugs (Yu et al., 2008). In this natural material-based experimental system, inorganic calcium carbonate is introduced into the vesicles formed using the FDA-approved drugs, protamine and heparin. Because the reaction medium is an aqueous solution, the entire preparation process contains no organic solvents; hence, the drug-carrying system lacks biological toxicity and improves cell and tissue compatibility.

Polymer-lipid hybrid nanovesicles have been explored as cancer drug delivery systems. Cheng et al. fabricated poly (ethylene oxide)-block-polybutadiene (PEO-PBD)-based nanovesicles, containing hydrogenated soy phosphatidylcholine and phospholipids (Cheng et al., 2011). Compared with simple polymer carriers, these nanovesicles have shown good anticancer effects *in vitro* and *in vivo*. Such hybrid vesicles combine the advantages of liposomes and vesicles and may improve drug-loading efficiency and reduce adverse effects (Discher et al., 1999; Discher et al., 2002; Ghoroghchian et al., 2005; Phan et al., 2021). Coleman et al. synthesized matrix metalloproteinase-9 (MMP-9)-cleavable lipopeptide PEGylated nanovesicles. *In vitro*, these nanovesicles promoted slow drug release in pancreatic ductal cancer cell models and improved antitumor efficacy when delivering gemcitabine. MMP-9 plays an important role in pancreatic cell carcinoma (Coleman et al., 2013), and the PEG-based nanovesicles of MMP-9 represent a potential new option for radical treatment of pancreatic cancer. Li et al. fabricated oxaliplatin-loaded carboxylatopillar-based pH-responsive nanovesicles. Compared with the free-drug group, the oxaliplatin-loaded vesicles improved the tumor inhibition efficiency by 70%. Host-guest nanovesicles were also used to (Li et al., 2017) simultaneously carry DOX and oxaliplatin. Within 24 h, the release of both drugs at pH 7.4 was ∼10% of that at pH 5.0, and *in vivo* experiments with a HepG-2 tumor model confirmed the safety of this drug delivery system (Chen et al., 2020). Xiao et al. developed copper-zeolitic imidazolate/carboxylated pillar six arene/methylene blue hybrid nanovesicles combining photodynamic and anticancer drug carriers. Cisplatin-loaded superparamagnetic iron oxide/perfluorohexane/silicate-polyaniline nanovesicles showed near-infrared stimulated cisplatin release and favorable antitumor effects (Xiao et al., 2021) (Figure 4).

#### 4.2.3 Polymer-Based Nanovesicle Delivery in Conjunction With Immunotherapy

Recent studies have explored nanovesicles as carriers combined with immunotherapy for treating malignant tumors. Exosome-mimetic nanovesicles carrying anti-programmed cell death ligand 1 (PD-L1) and CD73 inhibitors (AB680) effectively treated bladder cancer in a rat model. In bladder cancer, the immune escape of tumor cells remains a problem, and combined immunotherapy is a promising treatment strategy (Kavanagh et al., 2008). Introduction of AB680 in this experiment counteracted the high CD73 expression caused by anti-PD-1/PD-L1 treatment, thereby improving the effect of the immunotherapy (Allard et al., 2013). Corresponding animal experiments showed that the drug-loading system reduced the off-target effect of combined immunotherapy and improved the synergistic effect of combination therapy (Figure 3). DOX-loaded nanovesicles prepared from tumor-derived exosomes combined with liposomes were used in combination with immune checkpoint therapy and achieved long-term tumor-free survival in approximately 1/3 of mice (Mei Hu et al., 2021). Yu et al. developed a facile strategy to construct robust daratumumab immunopolymersomes (Dar-Ips). These can mediate safe CD38-targeted delivery of vincristine sulfate (Dar-IPs-VCR). Dar-IPs-VCR was constructed by postmodification via strain-promoted click-reaction holds with tailored antibody densities (2.2, 4.4 and 8.7 Dar per IP), superb stability, efficacious VCR loading, and glutathione-responsive VCR release. Dar4.4-IPs-VCR showed exceptional anti-MM activity against CD38-positive LP-1 MM cells and 12-fold and 20-fold enhancement compared with that of nontargeted IPs-VCR and free VCR controls in bare bone marrow and organ damage, respectively (Yu et al., 2021).

Choo et al. developed M1-macrophage-based vesicles (M1NVs) in conjunction with anti-PD-L1 antibodies. M1NVs can repolarize M2 TAM to M1 macrophages and showed potential antitumor efficacy for immunotherapy (Choo et al., 2018). Jung et al. prepared vesicle-coated PLGA microspheres (MS-VE) and encapsulated monophosphoryl lipid A (MPLA) (M/MS-VE). Compared with free MPLA, M/MS-VE triggered greater release of nine immune-stimulating cytokines, interleukin-6 (IL-6) and tumor necrosis factor-α (TNF-α), from ten macrophages and dendritic cells. This MS-VE could serve as a platform system for delivering immune stimulators and antigens to antigen-presenting cells with negligible toxicity. RNA interference (RNAi) combined with immunogenic chemotherapy can elicit potent antitumor immunity against cancer cells (Jung et al., 2019).

#### 4.2.4 Polymer-Based Biomacromolecule Vesicle Delivery System

Nanovesicle delivery systems are one of the most extensively studied drug carriers because they are cost-effective and reduce adverse reactions. Compared with viral vectors, polymer nanovesicles can protect biomacromolecules from degradation, prolong their time in circulation, promote target cell recognition and improve cellular uptake and intracellular escape.

Cationic polymer-lipid hybrid nanovesicles (P/LNVs) were developed as a novel delivery system for DOX and siRNA with broad cytotoxicity and gene-silencing efficiency against B16 cells. DOX-loaded P/LNVs directly increased the expression and presentation of endogenous tumor antigens *in situ* by inducing immunogenic cell death in B16 cells via the poly (ADP-ribose) polymerase 1-dependent (PARP1) apoptotic pathway. This resulted in a significant antitumoral immune response in mice. Using dying B16 cells as a vaccination strategy combined with RNAi-based knockdown of PD-L1 has shown efficacy in both preventive and metastatic melanoma. Notably, PD-L1 blockade acting synergistically with subtherapeutic doses of DOX triggered a robust therapeutic antitumoral T-cell response and eradicated pre-established tumors in 30% of mice with B16 melanoma. That study demonstrated that this combination therapy may provide a powerful new immunotherapeutic modality characterized by a significant increase in effector CD8^+^ T-cell infiltration and effective alleviation of the immunosuppressive tumor microenvironment (Wang et al., 2021). Ghaffari et al. exploited PEGylated niosomes loaded with miRNA-15a and miRNA-16-1 and the transfection prostate cell line, PC3. Transfecting PC3 cells with the nanocarriers loaded with the two miRNAs significantly decreased Bcl-2 gene expression and increased the degree of cell death in PC3 cells compared with those of the controls (Ghaffari et al., 2021).

Cell division cycle 20 homolog (CDC20) is an anaphase-promoting complex activator and a vital regulatory protein in the cell-cycle checkpoint. CDC20 overexpression is reported to promote the development of colorectal, pancreatic, non-small cell lung, and gastric cancers. Hemati et al. designed a niosome-encapsulated co-delivery system using DOX, quercetin and CDC20 siRNA. It showed thermosensitive drug-release behavior that successfully silenced the CDC20 expression compared with single delivery of siRNA or the drug. Moreover, the co-delivery of drugs and CDC20 siRNA strongly inhibited gastric cancer cell growth (Hemati et al., 2019).

### 4.3 Bioengineering Polymer-Based Nanovesicle Delivery System

Bioengineering technology has seen great progress and is widely used in anticancer treatment. Macrophage-based vesicles based on biopolymers prepared via bioengineering methods are also used to treat cancer, and lung metastasis can be treated through self-targeting (Cao et al., 2018). Self-assembled protein-polymer nanovesicles have been fabricated via *in situ* growth methods. Gao et al. developed self-growing nanovesicles of human serum albumin, which can deliver specific proteins and are expected to become a new drug carrier for cancer therapy (Liu and Gao, 2017). Liposome self-assembling materials are also used to prepare vesicles, and encapsulation of probe molecules and release of peptide bilayers have confirmed the feasibility of these materials as drug carriers, which are expected to be applied in antitumor therapy (Fatouros et al., 2014). DOX-loaded exosome-mimetic vesicles have similar therapeutic effects to those loaded with exosome-derived vesicles while significantly reducing adverse effects (Jang et al., 2014).

Immune evasion is a major obstacle facing T-cell tumor immunotherapy. Underexpression of tumor-rejection antigens leads to intrinsic immune resistance and interferon gamma (IFN-γ)-induced high expression of PD-L1 to further induce immune resistance. Dynemicin with DOX can effectively inhibit autophagy and increase major histocompatibility complex class I (MHC-I) levels in tumor cells. Zhou and Gao designed chameleon-mimetic prodrug nanovesicles for tumor-targeted delivery of DOX. These prodrug nanovesicles have a sheddable polyethylene glycol shell layer and CRGDK ligands, which remain stable during circulation while exposing targeting ligands in tumors, significantly inhibiting autophagy and inducing MHC-I expression, increasing tumor antigen presentation, recruiting more tumor-infiltrating T lymphocytes, and suppressing IFN-γ-induced intratumoral PD-L1 expression. After demonstrating the ability of these prodrug nanovesicles to overcome both intrinsic and induced immune evasion, the efficacy of the prodrug nanovesicles for cancer immunotherapy was experimentally validated in two tumor-bearing mouse models. This study may provide a new targeting strategy for reducing tumor immune resistance and enhancing tumor immunotherapy (Fengqi Zhou et al., 2021).

## 5 Conclusions and Perspectives

Drug delivery is an important application of polymer nanovesicles. Knowledge of cancer nanomedicine has drastically improved in recent decades. However, most approved nanomedicines have used existing drugs as payloads, and the use of small molecules for chemotherapy remains unsatisfactory. This review summarized the advances in polymer nanovesicles from the aspects of synthesis, preparation, multifunctions and applications. Polymeric nanovesicles have unique advantages over other polymersomes. Their specialized structures and properties exhibit good stability and permeability, easy functionalization, and smart stimulus responsiveness. These advantages make them one of the most promising supramolecular structures for potential applications in delivering drugs, genes and other therapeutic substances. Additional applications in immunotherapy, especially biomedical applications such as drug delivery and gene therapy, should be explored to promote the development of polymer nanovesicles.
